# Simulation of Radiative Transfer Within X-ray Microcalorimeter Absorbers

**DOI:** 10.1007/s10909-022-02754-4

**Published:** 2022-06-07

**Authors:** M. Lorenz, C. Kirsch, P. Peille, R. Ballhausen, V. Fioretti, S. Lotti, T. Dauser, J. Wilms

**Affiliations:** 1 Friedrich-Alexander-Universität Erlangen-Nürnberg, Dr. Karl Remeis-Observatory & ECAP, Sternwartstr. 7, 96049 Bamberg, Germany; 2 CNES, 18 Av. Édouard Belin, 31401 Toulouse Dedex 9, France; 3 Department of Astronomy, University of Maryland, College Park, MD 20742 USA; 4 Astrophysics Science Division, NASA-GSFC/CRESST, Greenbelt, MD 20771 USA; 5 INAF OAS Bologna, Via P. Gobetti 93/3, 40129 Bologna, Italy; 6 INAF IAPS, Via fosso del Cavaliere 100, 00133 Roma, Italy

**Keywords:** Simulations, Microcalorimeters, Transition-edge sensors

## Abstract

We present Monte Carlo simulations of radiative transfer within the absorbers of X-ray microcalorimeters, utilizing a numerical model for the photon propagation and photon absorption process within the absorber structure. In our model, we include effects of Compton scattering off bound electrons and fluorescence. Scattered or fluorescence photons as well as Auger and photoelectrons escaping the absorber can result in partial energy depositions. By implementing a simplified description of the physical processes compared to existing comprehensive particle transport software frameworks, our model aims to provide representative results at a small computational effort. This approach makes it possible to use our model for quick assessments, parametric studies, and application in other Monte Carlo-based instrument simulators like SIXTE, a software package for X-ray astronomical instrumentation. To study the impact of the energy loss effects on the spectral response of a microcalorimeter, we apply our model to the sensors of the cryogenic X-ray spectrometer X-IFU onboard the future *Athena* X-ray observatory.

## Introduction

Cryogenic X-ray microcalorimeters enable nondispersive spectroscopy with high energy resolution by sensing the temperature rise of an absorbing layer resulting from the interaction with incident photons and thermalization of their deposited energy [1]. Ideally, the energy of a photon is completely thermalized within the absorber structure. However, several energy loss mechanisms can reduce the total energy deposition and produce additional features and nonlinearities in the spectral response [2]. Escaping scattered photons deposit only a fraction of their energy within the absorber. Emitted fluorescence photons that are not re-absorbed lead to the formation of escape peaks. Auger and photoelectrons leaving the absorber surface produce a weak electron-loss continuum.

In this paper, we present a Monte Carlo model to predict the total energy deposition of incident photons within the absorbers of X-ray microcalorimeters, taking into account these energy loss mechanisms. The model is developed within the SIXTE (SImulation of X-ray TElescopes) software package^1^ [3], a Monte Carlo simulation toolkit for X-ray astronomical instrumentation, and xifusim [4], a dedicated simulator for the X-IFU microcalorimeter instrument [5] of the future *Athena* X-ray observatory [6]. Our model will be made available in a future release version of the SIXTE package.

Compared to existing general purpose, but computationally expensive, particle transport codes, as the Geant4 toolkit [7], the goal of our model is to provide representative distributions with minimal overhead and fast computation times. The model emerged from the need for a more realistic description of the involved physical processes in the SIXTE and xifusim simulators while keeping the impact on run time minimal. By applying several simplifications where appropriate for our problem domain, the model allows fast assessments of absorber properties. Our model enables, for example, parametric studies to aid in understanding how different characteristics result in different performances, which is essential to optimize the detector system for all intended applications.

Section 2 describes the simulation setup and implementation details. In Sect. 3, we show example results of our model and compare our output to a similar setup in the Geant4 framework.

## Model Description

**Fig. 1:**
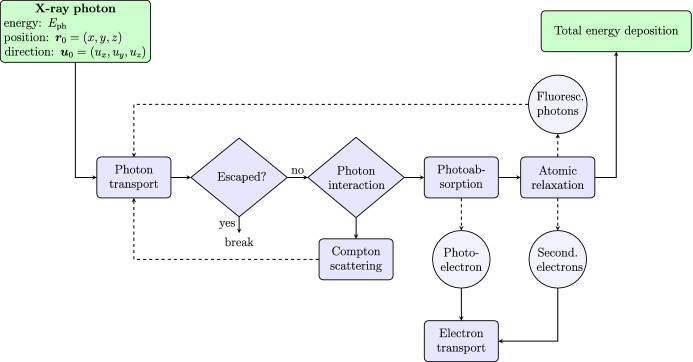
Flowchart of the radiative transfer model. Starting from a photon with energy $E_\mathrm {ph}$ and direction $\varvec{u}_0$ impacting the absorber structure at position $\varvec{r}_0$, we first propagate the photon through the absorber layers and sample an interaction position. In our simulation we include numerical models for photoelectric absorption, Compton scattering, atomic relaxation and electron transport. The output of our model is the total energy deposition within the absorber, taking energy loss due to escaping photons and electrons into account (Color figure online)

The input of our model is an X-ray photon with energy $E_\mathrm {ph}$ and initial direction $\varvec{u}_0=(u_x, u_y, u_z)$ impacting the absorber structure at position $\varvec{r}_0=(x,y,z)$. The output of the simulation is the total energy deposition within the absorber. We assume a rectangular absorber composed of several layers of different materials and thicknesses and apply a Cartesian coordinate system in which the z-axis points upward, and the xy-plane lies within the top surface of the absorber. We use photoelectric subshell cross sections from the Livermore Evaluated Photon Data Library [8–10]. Atomic relaxation information and transition probabilities are taken from the Livermore Evaluated Atomic Data Library (EADL) [11, 12]. Our model adopts principles of general Monte Carlo particle transport methods [13]. Figure 1 shows the flowchart of our radiative transfer simulation. The following subsections provide a summary of the individual simulation steps and our assumptions.

### Photon Transport

First, we propagate the photon through the absorber layers and calculate an interaction position, assuming straight-line trajectories in-between interactions. The optical depth $\tau$, the photon travels to its next interaction, is sampled from an exponential distribution^2^ [14]. To convert $\tau$ to the corresponding physical distance *l*, we solve the integral equation

1 $$\begin{aligned} \tau = \int _{0}^{l} \! \sigma _\mathrm {tot}(s)n(s) \, \mathrm {d}s\, \end{aligned}$$

along the photon path for *l*, where *n* is the number density of the layer materials. The total interaction cross section $\sigma _\mathrm {tot}$ is the sum of all subshell photoelectric absorption cross sections and total Compton scattering cross section at the given photon energy. Since both, *n* and $\sigma _\mathrm {tot}$, are constant within each layer, the corresponding physical distance *l* can directly be obtained from Eq. 1. If the photon escapes the absorber, the tracing of this photon stops. Otherwise, we propagate the photon to the next interaction position.

### Photon Interaction

In our simulation, we include numerical models for Compton scattering off bound electrons and photoelectric absorption. The interaction type is sampled based on the cross sections of these processes.

In the case of Compton scattering, we sample the energy $E'_\mathrm {ph}$ and the direction of the scattered photon from the Klein-Nishina differential cross section [15]. We incorporate incoherent scattering factors [15] to account for binding effects which also requires us to obtain the total Compton scattering cross section for a given incoming photon energy $E_\mathrm {ph}$ by numerical integration over all solid angles. The energy difference $E_\mathrm {ph}-E'_\mathrm {ph}$ is assumed to be deposited locally, and the scattered photon starts again at the photon transport step.

In the process of photoelectric absorption, a photoelectron is emitted from the corresponding subshell *i*, where the ionization occurs, with initial energy

2 $$\begin{aligned} E_\mathrm {e} = E_\mathrm {ph} - E_\mathrm {b,i}, \end{aligned}$$

where $E_\mathrm {b,i}$ is the binding energy of subshell *i*. For the angular distribution of the photoelectron emission we assume a uniform azimuthal angle relative to the photon direction. The polar angle is sampled from the Sauter-Gavrila distribution for the K-shell [16], as it is also done in the Geant4 package. The resulting vacancy triggers the atomic relaxation, while the photoelectron is followed up on with our electron transport model.

### Atomic Relaxation

The excited atom with an initial vacancy in the *i*-th subshell decays to its ground state through a series of radiative and nonradiative transitions. To sample a specific transition, we use subshell transition probabilities and corresponding energies of emitted fluorescence photons and Auger electrons provided by the Livermore EADL [11, 12]. New vacancies are followed up on recursively until all vacancies have moved to the outer subshells. The energy of the residual ion is deposited at the interaction position. We assume an isotropic emission of the fluorescence photons and Auger electrons and continue their propagation with the photon and electron transport stages.

### Electron Transport

To speed up the simulation process, we do not implement a comprehensive model of the electron scattering and thermalization within the absorber. Instead, we follow a simplified approach proposed in [17] to describe the electron transport, which also accounts for losses due to escaping electrons. The model utilizes the extrapolated projected electron range $R_\mathrm {ex}$ (in cm) [18]

3 $$\begin{aligned} R_\mathrm {ex}=\frac{A}{Z\rho }\exp \left( -4.5467-0.31104\ln {E_\mathrm {el}} + 0.07773\,(\ln {E_\mathrm {el}})^2\right) \times 10^{-6}, \end{aligned}$$

where *A* is the mass number of the absorbing medium, *Z* the atomic number, $\rho$ the density in $\mathrm {g}\,\mathrm {cm}^{-3}$, and $E_\mathrm {el}$ the energy of the electron in eV. Assuming straight-line electron tracks of range $R_\mathrm {ex}$, we consider the complete energy of the electron to be deposited if the electron stays within the absorber. If the electron reaches the absorber surface, we consider it to be escaped and calculate the deposited energy by integrating the effective stopping power [18]

4 $$\begin{aligned} \frac{\mathrm {d}E}{\mathrm {d}R_\mathrm {ex}}=\frac{E_\mathrm {el}}{R_\mathrm {ex}\left( 0.15546\ln {E}+0.31104 \right) } \end{aligned}$$

numerically along the track length inside the absorber. Equation 4 uses the same units as Eq. 3.

## Simulation Results

### Athena X-IFU Absorbers

As an application example, we show simulation results of our model for the Au/Bi absorbers of the *Athena* X-IFU Transition-Edge Sensor (TES) [19, 20] microcalorimeters. The X-IFU instrument is a high-resolution cryogenic X-ray spectrometer onboard the future *Athena* space X-ray observatory, operating a large array of Mo/Au-based TESs in the energy range from 0.2 to 12 keV with a spectral resolution of 2.5 eV FWHM up to 7 keV. In the current baseline configuration the absorber dimensions are 269.5 $\mu \mathrm {m}$ in width and length, composed of layers 0.04  $\mu \mathrm {m}$ Au, 5.15 $\mu \mathrm {m}$ Bi, and 1.22 $\mu \mathrm {m}$ Au (from top to bottom).

**Fig. 2:**
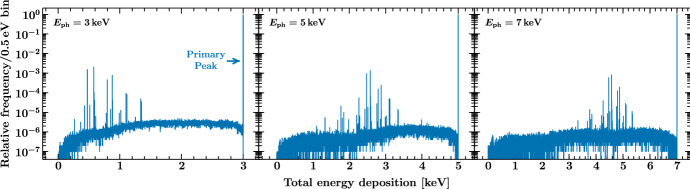
Distributions of the simulated total energy depositions within the *Athena* X-IFU absorber for different incident photon energies. We simulate $10^7$ events for each case, impacting perpendicularly and uniformly across the absorber surface. The sum of the relative frequencies in each case is equal to one. Our model produces escape peaks and the electron-loss continuum in the response below the primary peaks at $E_\mathrm {ph}$ (Color figure online)

**Fig. 3:**
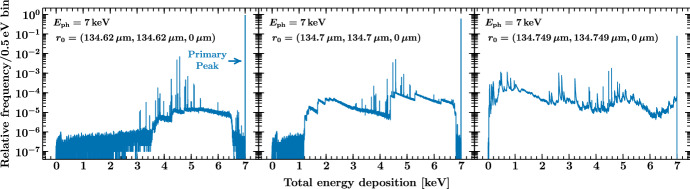
Same as Fig. 2, but for different impact positions near the absorber corner. The position (0, 0, 0) corresponds to the center of the absorber top and the dimensions of the absorber are $269.5\,\mu \mathrm {m} \times 269.5\,\mu \mathrm {m} \times 6.41\,\mu \mathrm {m}$. The electron-loss continuum increases close to the corner since electron escape becomes more probable. Escaping photoelectrons give also rise to edge-like features in the response. These edges arise whenever photoelectrons of a new subshell can contribute to the total energy deposition and are located at the corresponding binding energies, plus a small shift due to the deposited energy along the electron track inside the absorber. These features also become more prominent for impacts close to the absorber corner, where an escape is more likely to occur. The relative heights of the edges correspond to the subshell photoelectric cross sections at the given photon energy (Color figure online)

Figure 2 shows the distribution of the total energy depositions of $10^7$ incident photons for different initial energies, impacting perpendicularly and uniformly across the top of the absorber. We assume that each photon undergoes at least one interaction. The electron-loss continuum due to escaping Auger and photoelectrons emerges below the primary photon energies. Several distinct escape peaks are visible in the response. From the simulated event lists we obtain a percentage of events with only a partial energy deposition of about 1.7 % for 3 keV, 1.0 % for 5 keV, and 0.9 % for 7 keV incident photons. On average, higher energy photons penetrate deeper into the absorber, and the probability of photon and electron escape processes decreases.

To investigate the effect of the impact position on the resulting energy distribution, we repeat the simulation for different impact positions near the corner of the absorber. As shown in Fig. 3, the electron continuum and strength of the escape peaks increase for impacts near the corner as electron and photon escape is more likely to occur. For events very close to the corner of the absorber, the response is severely affected by the electron loss. The fraction of events with only a partial energy deposition is about 7.6 % for $\varvec{r}_0 = (134.62\,\mu \mathrm {m}, 134.62\,\mu \mathrm {m}, 0\,\mu \mathrm {m})$, 40 % for $\varvec{r}_0 = (134.7\,\mu \mathrm {m}, 134.7\,\mu \mathrm {m}, 0\,\mu \mathrm {m})$, and 92 % for $\varvec{r}_0 = (134.749\,\mu \mathrm {m}, 134.749\,\mu \mathrm {m}, 0\,\mu \mathrm {m})$.

### Comparison with Geant4 Simulations

**Fig. 4:**
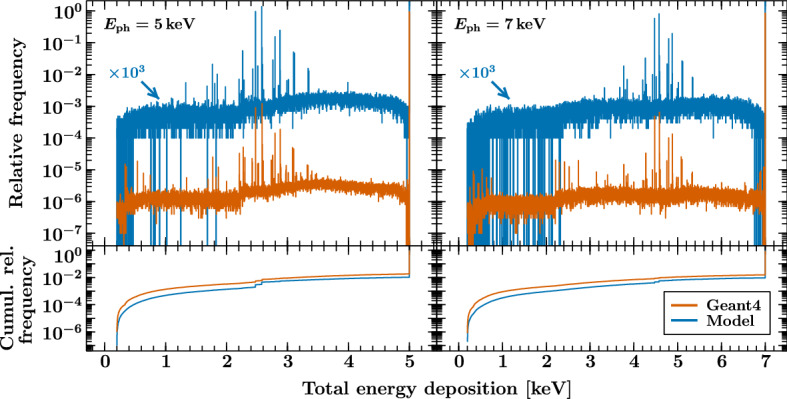
The top panels show a comparison between a Geant4 simulation of the full array of the *Athena* X-IFU absorbers and our model of a single absorber. The result of our model is scaled by a factor of $10^3$ to allow for easier comparison. The histograms are binned using one of the latest *Athena* X-IFU ancillary response files. The bottom panels compare the cumulative sums of the relative frequencies obtained with the Geant4 package and our model. Generally, the resulting escape peaks and continuum match well. The electron-loss continuum is slightly stronger in the Geant4 simulation. Additional emission features in the Geant4 data, emerging from escaping photons detected in neighboring pixels, are yet missing in our calculation (Color figure online)

To cross-check this approach, we have also compared the results of our model to distributions obtained with the well-established Geant4 package where we simulate the full array of the *Athena* X-IFU absorbers. Figure 4 shows the distribution of total energy depositions within the array resulting from $10^7$ uniformly distributed incident photons with initial energies of 5 and 7 keV. We also include the result of our model, scaled by a factor of $10^3$ to make it easier to compare the data, and the cumulative sum of the relative frequencies obtained with both models.

In general, we find a good agreement between the two models. Our approach predicts escape peaks of similar strength, and the electron-loss continua follow comparable shapes, where the overall intensity is slightly higher in the Geant4 simulation. While the Geant4 toolkit provides extensive models of the involved physical processes, our simplified approach seems well-suited to provide representative distributions. Considering the savings in computation time from several hours of the Geant4 setup to about 30 seconds with our model to simulate the $10^7$ events in this case, both approaches can also complement each other depending on the goals of the study.

## Conclusions

We have presented a Monte Carlo model to predict the total energy deposition of incident photons within X-ray microcalorimeter absorbers. Our model reproduces characteristic features in the spectral response like escape peaks and the electron-loss continuum. Comparisons of our simplified model with comprehensive Geant4 absorber simulations show a good agreement of the resulting distributions. Given the short run time of our model, this approach is well-suited for application in parametric studies and to enhance X-ray microcalorimeter models in other Monte Carlo-based simulators like SIXTE and xifusim with only a small overhead. As one of the following steps, we will compare our results with measured data. We also want to further investigate the slight remaining difference to the Geant4 simulation and refine our model accordingly. We also plan to include a more accurate treatment of the thermalization mechanism if possible without negatively impacting the run time.

## Footnotes

1 https://www.sternwarte.uni-erlangen.de/research/sixte/.

2 Strictly, the parameter sampled as path length in optical depth space follows the same distribution as the geometrical penetration depth in a homogeneous medium.
